# A parallel method for enumerating amino acid compositions and masses of all theoretical peptides

**DOI:** 10.1186/1471-2105-12-432

**Published:** 2011-11-07

**Authors:** Alexey V Nefedov, Rovshan G Sadygov

**Affiliations:** 1 Department of Biochemistry and Molecular Biology, Sealy Center for Molecular Medicine, University of Texas Medical Branch, 301 University Blvd, Galveston, TX 77555, USA

## Abstract

**Background:**

Enumeration of all theoretically possible amino acid compositions is an important problem in several proteomics workflows, including peptide mass fingerprinting, mass defect labeling, mass defect filtering, and de novo peptide sequencing. Because of the high computational complexity of this task, reported methods for peptide enumeration were restricted to cover limited mass ranges (below 2 kDa). In addition, implementation details of these methods as well as their computational performance have not been provided. The increasing availability of parallel (multi-core) computers in all fields of research makes the development of parallel methods for peptide enumeration a timely topic.

**Results:**

We describe a parallel method for enumerating all amino acid compositions up to a given length. We present recursive procedures which are at the core of the method, and show that a single task of enumeration of all peptide compositions can be divided into smaller subtasks that can be executed in parallel. The computational complexity of the subtasks is compared with the computational complexity of the whole task. Pseudocodes of processes (a master and workers) that are used to execute the enumerating procedure in parallel are given. We present computational times for our method executed on a computer cluster with 12 Intel Xeon X5650 CPUs (72 cores) running Windows HPC Server. Our method has been implemented as a 32- and 64-bit Windows application using Microsoft Visual C++ and the Message Passing Interface. It is available for download at https://ispace.utmb.edu/users/rgsadygo/Proteomics/ParallelMethod.

**Conclusion:**

We describe implementation of a parallel method for generating mass distributions of all theoretically possible amino acid compositions.

## Background

Mass spectrometry (MS) plays a crucial role in modern proteomics as a key method for protein identification and quantification. MS provides accurate mass and abundance measurements of intact and fragmented peptide ions, which are then processed by specialized algorithms and transformed into peptide and protein identities. Thus, efficiency of many MS-based proteomics workflows depends on how well we understand -- and can utilize -- the properties of peptide masses and peptide mass distribution.

It has been observed that peptide masses have a nonuniform, clustered distribution, which is explained by the fact that peptides are made of twenty amino acids with specific masses. This distribution consists of repeating peaks separated by approximately 1 Da, which become taller and wider as the mass increases. Consecutive peaks are separated by low populated regions (quiet zones) and gaps (forbidden zones)-that is, the mass ranges for which there exist no possible sequences of amino acids. Nonuniformity (peaks, gaps) and discrete nature of the mass distribution of peptides are important for two major problems in MS-based proteomics: peptide identification and *de novo *sequencing.

The knowledge of the mass distribution of a particular type of peptide (for example, non-modified tryptic peptides) can be used to facilitate peptide identification in a number of ways. Forbidden zones allow us to filter out MS signals corresponding to non-target species (nonpeptide contaminants or modified peptides) early on, before doing any complicated processing of MS data. Dodds and coworkers [1] showed that this results in exponential improvements in statistical significance and discrimination of protein identification based on peptide mass fingerprinting on the Mascot platform. Nonoverlapping or partially overlapping peaks in the mass distributions of different types of peptides allow recognition of these types based solely on precursor masses. For example, Spengler and Hester [2] showed that accurate masses (with accuracy of 0.1 or even 1 ppm) allow phosphorylated and nonmodified peptides to be distinguished. Lehmann and coworkers [3] and Jones and coworkers [4] showed that this is possible for glycopeptides and lipids. In addition, there have been many suggestions for label tags shifting the mass of labeled peptides to quiet or forbidden zones in order to allow easier identification and quantification of these peptides [5].

The major drawback of peptide identification algorithms based on database search is their inability to identify peptides that are not present in the reference database. *De novo *sequencing algorithms are designed to restore peptide compositions from MS data without the use of peptide databases. These algorithms employ several strategies for MS data analysis s [6], one of which is based on the fact that for a given mass there exist only a finite (though sometimes very large) number of amino acid sequences (or amino acid compositions) that can assume that mass, and that these sequences (compositions) can be explicitly enumerated. The use of the masses of fragment ions can further reduce the number of admissible compositions. Several reports have shown the feasibility of this strategy, especially for high accuracy data provided by modern Fourier transform mass spectrometers [7-9].

Proteomics applications mentioned above rely on specific properties of the peptide mass distributions that can only be obtained by enumerating all theoretically possible peptides. Moreover, in many circumstances it is impossible to generate these distributions once and for all, as many parameters can vary from experiment to experiment (peptide modifications, enzymatic specificity, number of missed cleavages, etc.) Thus, it is desirable to be able to generate peptide mass distributions (or some parts of these distributions) "to order" and, therefore, to be able to generate them fast.

Several works focusing on different MS-based proteomics applications employed enumeration of all theoretically possible peptides [8,10-13]. Because of the high computational complexity of the task, enumeration of peptides was done for the mass range below 2 kDa, which limited applicability of the obtained results. Also, even for this mass range long computational times and extensive computational capabilities were often required. Olson and others [8] mentioned the use of a parallel method for peptide enumeration, but details of its implementation as well as its computational performance were not reported.

In a recent paper [14] we described the mass distribution of all theoretically possibly tryptic peptides made of 20 amino acids, up to the mass of 3 kDa. The paper provided detailed characterization of forbidden zones and amino acid compositions of peptides from the quiet zones. We showed how forbidden zones shrink over the mass range, where they completely disappear and how they depend on the measured mass accuracy. We found that peptide sequence compositions in the quiet zones are less diverse than those in the peaks of the distribution, and that forbidden zones may be extended by eliminating certain types of unrealistic compositions. We also characterized symmetry of mass peaks and the accuracy of the Mann's equations [13] for the mass peak position and width. Our study was made possible by advancing computational techniques for the enumeration of amino acid compositions.

In this paper, we describe in detail a parallel method for enumerating all amino acid compositions up to a given length. First, we present a pseudocode for recursive procedures which are the core of this method. We then show how a single task of enumerating all peptide compositions can be divided into smaller subtasks that can be executed in parallel. We also show how the computational complexity of these subtasks compares with the computational complexity of the primary task. Finally, we provide pseudocode of processes (a master and workers) that are used to execute the enumerating procedure in parallel. To the best of our knowledge this is the first description of a computational method for a complete and unbiased enumeration of all theoretically possible peptides. We present computational times for our method, implemented by using Microsoft Visual C++ and the Message Passing Interface (MPI), and executed on a computer cluster with 12 Intel Xeon X5650 CPUs running Windows HPC Server 2008. The mass and length limits are input parameters of the program.

## Implementation

### Peptide compositions

Any peptide composition is represented by a numerical vector (*n*_1_, *n*_2_,..., *n*_20_), whose i-th component is equal to the number of times the i-th amino acid occurs in the peptide. For example, sequence *a*_1_*a*_20_*a*_1_*a*_1 _has composition (3, 0,..., 0, 1). In some cases, it is convenient to consider peptides as sequences composed of less or more than 20 letters (tryptic peptides without missed cleavages, post-translationaly modified peptides, etc.). For this reason, let us adopt a more general notation: assume we have an alphabet of *N *characters and composition vectors (*n*_1_, *n*_2_,..., *n_N_*). The length of a composition is defined as *L *= *n*_1 _+ *n*_2 _+... + *n_N_*. If *m_i _*is the monoisotopic mass associated with the i-th letter, then the monoisotopic mass of a composition is defined as *m *= *n*_1_*m*_1 _+ *n*_2_*m*_2 _+... + *n_N_m_N _*(the monoisotopic mass of H_2_O and a proton may be added to this mass if necessary.)

For a single sequence of letters we have a uniquely defined composition, while for a single composition of length *L *we have

(1) $$\frac{L!}{n_{1}!n_{2}!\;...\;n_{N}!}$$

corresponding sequences, given by the multinomial coefficient. Note that all these sequences will have the same mass, which explains the convenience of enumerating peptide compositions instead of peptide sequences in order to obtain all theoretically possible peptide masses.

The number of compositions of length *L *is equal to the number of ways to choose *L *elements from a set of *N *elements if repetitions are allowed:

(2) $$\left(\begin{array}{c} N+L-1\\ L \end{array}\right)=\frac{\left(N+L-1\right)!}{L!\left(N-1\right)!}.$$

The number of compositions of all lengths not greater than *L *(including one composition of length 0) is equal to

(3) $$\overset{L}{\underset{k=0}{\sum }}\left(\begin{array}{c} N+k-1\\ k \end{array}\right)=\left(\begin{array}{c} N+L\\ N \end{array}\right)$$

which follows from the equation

$$\overset{n}{\underset{k=0}{\sum }}\left(\begin{array}{c} r+k\\ k \end{array}\right)=\left(\begin{array}{c} r+n+1\\ n \end{array}\right).$$

The latter is based on the recurrence relation

$$\left(\begin{array}{c} r-1\\ k \end{array}\right)+\left(\begin{array}{c} r-1\\ k-1 \end{array}\right)=\left(\begin{array}{c} r\\ k \end{array}\right),$$

and can be found in the book by Graham and others [15]. The number of sequences of all lengths not greater than *L *is equal to

$$\frac{N\left(N^{L}-1\right)}{N-1}.$$

Table 1 shows the number of compositions and sequences for peptides comprised of 20 amino acids. Note how the number of sequences exceeds the number of compositions as the length of peptides grows.

**Table 1:** Number of compositions and sequences comprised of 20 letters, of length not greater than *L*, for *L *ranging from 3 to 10, and their ratios (rounded)

Length of Peptides (*L*)	Number of Compositions (*A*)	Number of Sequences (*B*)	Ratio *B*/*A*
3	1,770	8,420	5

4	10,625	168,420	16

5	53,129	3,368,420	63

6	230,229	67,368,420	293

7	888,029	1,347,368,420	1,517

8	3,108,104	26,947,368,420	8,670

9	10,015,004	538,947,368,420	53,814

10	30,045,014	10,778,947,368,420	358,760

### Enumerating peptide compositions

**Figure 1:**
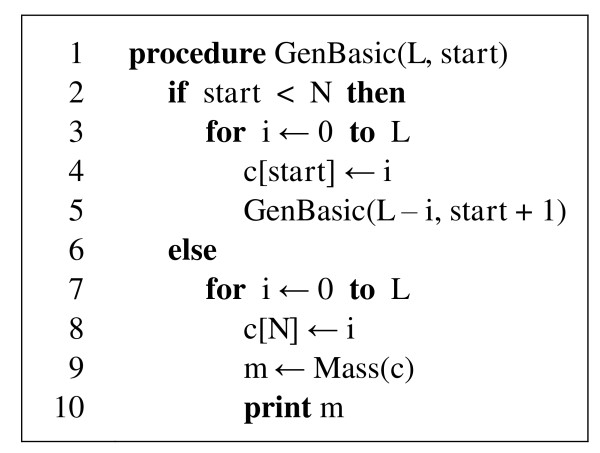
**Pseudocode for recursive procedure GenBasic which enumerates all compositions of length not greater than *L *and prints their masses**.

Figure 1 shows the pseudocode of a basic recursive procedure for enumerating all compositions of length not greater than *L *for an alphabet of *N *letters. Array *c *holds current composition (*n*_1_, *n*_2_,..., *n_N_*) and is indexed from one. Procedure Mass returns the mass of the input composition *c*. For a given length *L*, procedure GenBasic should be called with parameters (*L*, *start *= 1). Note that the depth of recursion for this procedure is equal to *N *- 1. The number of compositions enumerated by this procedure is given by equation (3).

Procedure GenBasic begins enumeration with composition (0, 0,..., 0) and first generate all compositions with *n_N _*ranging from 0 to *L*. It then sets *n*_*N*-1 _to 1, and generates all compositions with *n_N _*ranging from 0 to *L *- 1, and so on. The last composition in this generation process is (*L*, 0,..., 0). Essentially, the compositions are generated like *N*-digit numbers, in ascending order, with requirement that the sum of the "digits" must not be greater than *L*. For instance, for *N *= 3 and *L *= 2 the procedure generates all compositions up to length 2 in the following order: (0, 0, 0), (0, 0, 1), (0, 0, 2), (0, 1, 0), (0, 1, 1), (0, 2, 0), (1, 0, 0), (1, 0, 1), (1, 1, 0), (2, 0, 0).

Several changes to procedure GenBasic will make it faster. First, if *L *is equal to zero on line 3 then there is no need to make assignment on line 4 and call GenBasic on line 5, since it is already known that the rest of the composition will contain zeros only. Second, we can calculate the mass of a composition as soon as its component *n_i _*becomes known, and then pass this mass to the next call of the generating procedure. By doing this, we avoid the need to recalculate the mass of the part of the composition that has not been changed.

**Figure 2:**
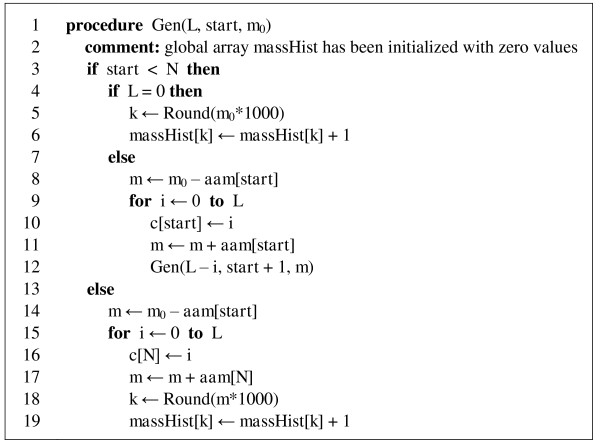
**Pseudocode for recursive procedure Gen, a faster version of GenBasic**. The procedure generates the mass histogram of all compositions of length not greater than *L*.

Figure 2 shows the pseudocode of procedure Gen which is a faster version of procedure GenBasic. It generates a histogram of peptide compositions' masses, instead of printing them, which is more suitable for its further use. The histogram, stored in global array *massHist*, contains the number of compositions falling into the mass bins of width 0.001 Da. Procedure Round returns the rounded integer value of its argument. Note that since procedure Gen calculates the mass of compositions "on the fly", we do not need to store compositions in array *c*, so lines 10 and 16 may be removed. We assume that array *aam *of size *N *stores masses *m*_1_, *m*_2_,..., *m_N _*. Procedure Gen should be called with parameters (*L*, *start *= 1, *m*_0 _= 0).

### Enumerating peptide compositions in parallel

**Figure 3:**
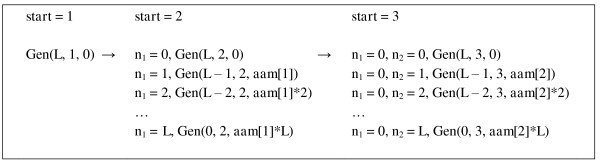
**Creating multiple jobs from a single job of enumerating all compositions**. A single call to procedure Gen with parameters (*L*, 1, 0) is equivalent to *L*+1 calls with parameters (*L*, 2, 0), (*L *- 1, 2, aam [1]),..., (0, 2, aam [1]*L), while *n*_1 _is set to 0, 1,..., *L*, correspondingly. Any job with *start *= 2 can be further expanded into *L*+1 jobs with *start *= 3, as shown for Gen(*L*, 2, 0).

The task of enumerating all compositions (*n*_1_, *n*_2_,..., *n_N_*) can be split into smaller independent subtasks or jobs that can be executed in parallel. Indeed, a single call to procedure Gen with parameters (*L*, 1, 0) is equivalent to *L*+1 calls with parameters (*L*, 2, 0), (*L *- 1, 2, *aam* [1]),..., (0, 2, *aam* [1]*L), while *n*_1 _is set to 0, 1,..., *L*, correspondingly (Figure 3). As before, we assume that array *aam *stores masses *m*_1_, *m*_2_,..., *m_N _*of the used amino acids. Certainly, we will have to combine mass histograms produced by each call of procedure Gen, which can be done knowing parameters of each job, described by a triplet (*L*, *start*, *m*_0_).

To illustrate this idea, consider again our example with *N *= 3 and *L *= 2. The primary task is to enumerate the following compositions: (0, 0, 0), (0, 0, 1), (0, 0, 2), (0, 1, 0), (0, 1, 1), (0, 2, 0), (1, 0, 0), (1, 0, 1), (1, 1, 0), (2, 0, 0). This can be accomplished by independent enumeration of three subsets of compositions: (i) (0, 0, 0), (0, 0, 1), (0, 0, 2), (0, 1, 0), (0, 1, 1), (0, 2, 0); (ii) (1, 0, 0), (1, 0, 1), (1, 1, 0); and (iii) (2, 0, 0). Compositions (i) can be enumerated by setting *n*_1 _= 0 and calling Gen with parameters (*L *= 2, *start *= 2, *m*_0 _= 0); compositions (ii) can be enumerated by setting *n*_1 _= 1 and calling Gen with parameters (*L *= 1, *start *= 2, *m*_0 _= *m*_1_); and single composition (iii) is enumerated by setting *n*_1 _= 2 and calling Gen with parameters (*L *= 0, *start *= 2, *m*_0 _= 2*m*_1_).

How can we create a list or table of jobs given the initial job described by parameters (*L*, 1, 0)? First, job (*L*, 1, 0) is replaced by *L*+1 jobs (*L*, 2, 0), (*L *- 1, 2, *aam*[1]),..., (0, 2, *aam* [1]*L) (Figure 3). If, for a given *L*, job (*L*, 2, 0) is executed in acceptable time, we do not need to do anything else, and the table of jobs has been initialized. Otherwise, we can split job (*L*, 2, 0) into *L*+1 jobs with *start *= 3, and similarly split other jobs with *start *= 2. Thus, for all jobs with *start *= 2 there is certain *L*_max,2 _such that if the first parameter of the job is larger than *L*_max, 2 _then this job should be split into jobs with *start *= 3. When this is done, we move to the jobs with *start *= 3 and process them in a similar manner: all jobs that have first parameter larger than *L*_max,3 _should be split into jobs with *start *= 4. We continue this until each job in the job table can be executed in acceptable time (see additional notes on this in the Discussion section).

**Figure 4:**
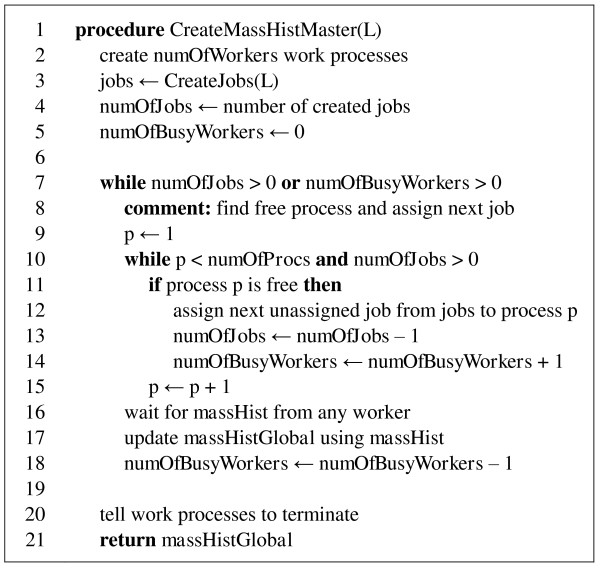
**Pseudocode for procedure CreateMassHistMaster**.

**Figure 5:**
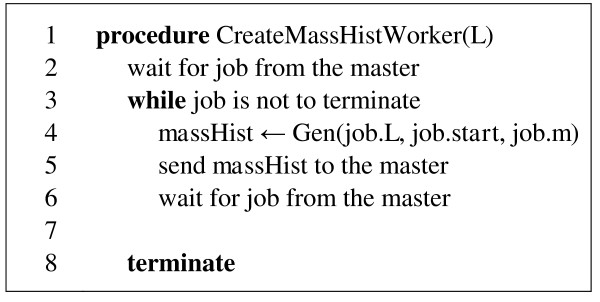
**Pseudocode for procedure CreateMassHistWorker**.

When the table of jobs has been initialized, the jobs from the table can be assigned to computation processes. In this context, it is convenient to think about a master process, which does these assignments (Figure 4), and worker processes (Figure 5), which execute the assigned jobs and return results back to the master. The master then combines partial mass histograms computed by workers into a single final mass histogram. There may be different strategies utilized in assigning the jobs. For example, larger jobs (with larger *L*) may be assigned prior to smaller jobs (with smaller *L*). In our experiments, which are presented in the Discussion section, there were no particular strategy in job assignments (jobs were assigned in the order in which they had been inserted into the job table).

The data exchange between the master and workers (Figure 4, lines 12, 16; Figure 5, lines 2, 5, 6) can be organized by using functions MPI_Send and MPI_Receive from any library implementing MPI [16]. In our implementation, we used Microsoft Visual C++ and MPI library from Microsoft HPC SDK Pack.

## Results and Discussion

It is worthwhile to make several additional comments on procedure Gen, presented on Figure 2. Various practical considerations may suggest using an upper limit on the mass of peptide compositions that one wants to enumerate. In this case, a significant improvement in computation speed may be achieved by canceling the enumeration of compositions whose mass exceeds a given limit. If array *aam *contains mass values in ascending order, we can return from function Gen as soon as the current mass (*m*_0 _in line 5, *m *in lines 11 and 17) exceeds the threshold. To illustrate a possible gain in speed that may be achieved by using a maximum mass limit, consider enumeration of compositions corresponding to all tryptic peptides up to the length of 30. It takes 1 hour 20 minutes to complete the full enumeration of such compositions, while with the mass limit of 3 kDa (heavier peptides are rarely identified in MS experiments) it takes only 11 minutes, as about 87% of the compositions can be skipped (Tables 2 and 3).

**Table 2:** Computation times for enumerating all tryptic compositions up to the length of 30, for different sets of jobs and number of work processes, with and without the maximum mass limit

Task	Number of Workers	Job Table					Computation Time	
		**Number** **of Jobs**	***start***	***L*_max,2_**	***L*_max,3_**	***L*_max,4_**	**massMax = 3 kDa**	**no massMax**

*L *= 30	1	1	1	-	-	-	6 h 03 min	35 h 11 min
	
	5	30	2	-	-	-	2 h 12 min	14 h 52 min
	
	30	30	2	-	-	-	1 h 39 min	13 h 32 min
	
	30	255	≤ 3	20	-	-	28 min	5 h 02 min
	
	71	255	≤ 3	20	-	-	27 min	4 h 57 min
	
	71	679	≤ 5	20	24	28	11 min	1 h 20 min

Computations were done using 71 work processes executed on a cluster with 12 Intel Xeon X5650 CPUs running Windows HPC Server 2008.

**Table 3:** Computation times for enumerating all tryptic compositions with different maximum lengths, with and without maximum mass limit

*L*	Computation Time	
	***maxMass *= 3 kDa**	**no mass limit**

25	19 min	29 min

30	11 min	1 h 20 min

35	8 min	5 h 38 min

40	8 min	38 h 28 min

45	14 min	> 96 h

50	29 min	-

Parameters of the job table were: *start *≤ 7, *L*_max,2 _= 20, *L*_max,3 _= 24, *L*_max,4 _= 28, *L*_max,5 _= 34, *L*_max,6 _= 40. Computations were done using 71 work processes executed on a cluster with 12 Intel Xeon X5650 CPUs running Windows HPC Server 2008.

There may be other modifications to this procedure, depending on the intended use of the generated mass distribution. For example, the maximum number of occurrences of each amino acid in a peptide may be made limited by a threshold based on the amino acid and the length and/or mass of the peptide. This would make the generated mass distribution more realistic and may increase the lengths of forbidden zones [14]. Instead of counting the number of peptide compositions, one can count the number of peptide sequences using equation (1). In this case, efficient computation of factorials "on the fly" can be implemented similar to the computation of peptide masses. If we are interested in enzyme-specific peptides, the procedure can be modified to allow a given number of missed cleavages. The number of amino acids (*N*) and their monoisotopic masses may vary depending on specific proteases used in sample preparation, possible post-translational or chemical modifications, and other factors. The resolution of the mass histogram (0.001 Da) may be changed as well, without significantly impairing computational speed.

An important question is how the job (*L*, *start *+ 1, 0) compares with the job (*L*, *start*, 0) in terms of computational complexity. Let us denote the number of compositions enumerated by the first procedure by *C*(*L*, *start *+ 1), and the number of compositions enumerated by the second procedure by *C*(*L*, *start*). Using equation (3) we have:

$$C\left(L,\;start\right)=\left(\begin{array}{c} N-start+1+L\\ N-start+1 \end{array}\right).$$

Hence,

$$\frac{C\left(L,\;start\right)}{C\left(L,\;start+1\right)}=1+\frac{L}{N-start+1}.$$

For example, if *N *= 20, *L *= 40, and *start *= 1, then *C*(40, 1)/*C*(40, 2) = 3, which means that we get a three-fold decrease in computation time by replacing one call Gen(40, 1, 0) by 41 calls to Gen with *start *= 2, executed in parallel. Similarly, we get

$$\frac{C\left(L,\;start\right)}{C\left(L-1,\;start\right)}=1+\frac{N-start+1}{L}.$$

Thus, if *N *= 20, *L *= 40, and *start *= 2, then *C*(40, 2)/*C*(39, 2) ≈ 1.5, which means that Gen(39, 2, 0) will be about 1.5 times faster than Gen(40, 2, 0).

Initialization of a job table requires the maximum value of parameter *start*, as well as parameters *L*_max,2 _, *L*_max,3 _, etc., to be specified. These can be determined empirically based on the available computational resources and the number of processes that can be executed in parallel. For example, we found that for enumerating tryptic peptide compositions of masses up to 3 kDa by using 72 processes running on 12 Intel Xeon X5650 CPUs the following parameters would give good performance: *start *≤ 7, *L*_max,2 _= 20, *L*_max,3 _= 24, *L*_max,4 _= 28, *L*_max,5 _= 34, *L*_max,6 _= 40. The tuning of these parameters is important to ensure good performance, as they directly affect the computation time (Table 2).

It should be noted that a job table may have jobs with the same parameters *L *and *start*, differing only in *M*. For example, consider the case illustrated in Figure 3. Splitting job (*L*, 2, 0) into *L*+1 jobs with *start *= 3 will give us, among others, job (*L-*1, 3, *aam*[2]). On the other hand, splitting job (*L-*1, 2, *aam*[1]) into *L *jobs with *start *= 3 gives us job (*L-*1, 3, *aam*[1]). It is clear that execution of these two jobs can be done in one call to function Gen, which should be modified to be able to accept two input masses $m_{0}^{1}$, $m_{0}^{2}$ instead of *m*_0_, and to work with two variables *m*^1^, *m*^2 ^instead of *m*. In a similar manner, execution of more than two jobs may be done in one call to function Gen. This approach will lead to a significant speed-up in computations (it has not been implemented in our code).

In fact, a job table may have jobs with all three parameters *L*, *start *and *M *being equal. Consider, for example, a primary job with *L *= 40, *start *= 0, and *m*_0 _= 0. Assume that array *aam *holds amino acid masses in ascending order. Then the first five masses stored in this array will correspond to glycine (G), alanine (A), serine (S), proline (P) and valine (V), and we can denote the first five elements of a composition by *n_G_*, *n_A_*, *n_S_*, *n_P_*, *n_V_*. Assume that the job splitting algorithm (see subsection 2.3) yields the following two jobs:

$$\begin{array}{c} n_{G}=2,n_{A}=0,n_{S}=0,n_{P}=0,n_{V}=1,start=6,L=37,\\ n_{G}=0,n_{A}=3,n_{S}=0,n_{P}=0,n_{V}=0,start=6,L=37. \end{array}$$

Then these two jobs will have the same *m*_0 _= 213.111 Da, since tripeptides GGV and AAA are isomeric. If a job table is generated using parameters *start *≤ 7, *L*_max,2 _= 20, *L*_max,3 _= 24, *L*_max,4 _= 28, *L*_max,5 _= 34, *L*_max,6 _= 40, then for *L *= 40 about 2% of all jobs will be duplicates; for *L *= 50 -- about 29%, and for *L *= 60 -- about 47%. In the case when we are only interested in the mass distribution of peptide compositions, there is no need to execute duplicate jobs. If certain job occurs *k *times, it is enough to execute it once and then multiply the resulting histogram by *k *before adding it to the final histogram. However, if we would like to get every peptide composition, then we cannot remove duplicate jobs.

In the end of this section, we present Table 3 which shows computation times for enumeration of tryptic compositions for a range of lengths between 25 and 55, with and without the use of a maximum mass limit. The numbers in the second column may seem counterintuitive at first, since, for example, it takes 19 min to generate the distribution for *L *= 25 and 11 min for *L *= 35. The explanation, however, lies in using the maximum mass limit of 3 kDa. The longest job for the task with *L *= 25 was *L *= 24, *start *= 2, *m*_0 _= 0, and it executed for 19 min. The longest job for the task with *L *= 30 was *L *= 24, *start *= 2, *m*_0 _= 285, and it executed for 8 min. The difference in 11 min comes from the fact that more compositions were canceled out in the second case because of the mass limit that was used.

We would like to note, in addition to Table 3, that enumeration of all tryptic peptides having the mass no greater than 3 kDa (the length of these peptides does not exceed 51) took 32 minutes.

## Conclusions

In this paper, we presented a detailed description of a parallel method for enumerating all theoretically possible amino acid compositions and discussed different aspects of its implementation. Enumeration of all amino acid compositions is important in several proteomics workflows, including peptide mass fingerprinting, mass defect labeling, mass defect filtering, and de novo peptide sequencing. Given the fact that multi-core computers and computer clusters are becoming increasingly available, it is natural to address this computationally expensive task using a parallelization approach.

We believe that by reducing computational times from hours to minutes, the applicability of the enumeration of all amino acid compositions in various proteomics studies may be significantly improved and extended. We have used the method described in this work to characterize forbidden and quiet zones in the mass distribution of tryptic peptides [14]. In the next step, we plan to apply this method to enhance the accuracy of protein identification in real mass spectrometry data. Our method has been implemented as a 32- and 64-bit Windows application using Microsoft Visual C++ and MPI. It is freely available for download at https://ispace.utmb.edu/users/rgsadygo/Proteomics/ParallelMethod.

### Availability and Requirements

**• Project Name: **PepComp

**• Project home page: **https://ispace.utmb.edu/users/rgsadygo/Proteomics/ParallelMethod

**• Operating System**: MS Windows

**• Other Requirements**: Message Passing Interface, multi-core CPU

**• Programming Language**: Visual Studio C++

**• License**: No license needed

## Competing interests

The authors declare that they have no competing interests.

## Authors' contributions

RGS conceived the project. AVN conducted the analysis. AVN and RGS wrote the paper. All authors contributed to the underlying ideas of the method and the analysis. All authors read and approved the final manuscript.
